# Assessment of the impact of EHR heterogeneity for clinical research through a case study of silent brain infarction

**DOI:** 10.1186/s12911-020-1072-9

**Published:** 2020-03-30

**Authors:** Sunyang Fu, Lester Y. Leung, Anne-Olivia Raulli, David F. Kallmes, Kristin A. Kinsman, Kristoff B. Nelson, Michael S. Clark, Patrick H. Luetmer, Paul R. Kingsbury, David M. Kent, Hongfang Liu

**Affiliations:** 1 Department of Health Sciences Research, Mayo Clinic, Rochester, MN USA; 2 Department of Neurology, Tufts Medical Center, Boston, MA USA; 3 Department of Radiology, Mayo Clinic, Rochester, MN USA; 4 Institute for Clinical Research and Health Policy Studies, Tufts Medical Center, Boston, MA USA

**Keywords:** Electronic health records, Reproducibility, Clinical research informatics, Data quality, Multi-site studies, Learning health system

## Abstract

**Background:**

The rapid adoption of electronic health records (EHRs) holds great promise for advancing medicine through practice-based knowledge discovery. However, the validity of EHR-based clinical research is questionable due to poor research reproducibility caused by the heterogeneity and complexity of healthcare institutions and EHR systems, the cross-disciplinary nature of the research team, and the lack of standard processes and best practices for conducting EHR-based clinical research.

**Method:**

We developed a data abstraction framework to standardize the process for multi-site EHR-based clinical studies aiming to enhance research reproducibility. The framework was implemented for a multi-site EHR-based research project, the ESPRESSO project, with the goal to identify individuals with silent brain infarctions (SBI) at Tufts Medical Center (TMC) and Mayo Clinic. The heterogeneity of healthcare institutions, EHR systems, documentation, and process variation in case identification was assessed quantitatively and qualitatively.

**Result:**

We discovered a significant variation in the patient populations, neuroimaging reporting, EHR systems, and abstraction processes across the two sites. The prevalence of SBI for patients over age 50 for TMC and Mayo is 7.4 and 12.5% respectively. There is a variation regarding neuroimaging reporting where TMC are lengthy, standardized and descriptive while Mayo’s reports are short and definitive with more textual variations. Furthermore, differences in the EHR system, technology infrastructure, and data collection process were identified.

**Conclusion:**

The implementation of the framework identified the institutional and process variations and the heterogeneity of EHRs across the sites participating in the case study. The experiment demonstrates the necessity to have a standardized process for data abstraction when conducting EHR-based clinical studies.

## Background

The rapid adoption of electronic health records (EHRs) holds great promise for transforming healthcare with EHR enabled continuously learning health systems (LHS), first envisioned by the Institute of Medicine in 2007 [1]. A continuously learning health system can enable efficient and effective care delivery with the ability to discover practice-based knowledge and the seamless integration of clinical research with care practice [2, 3]. To achieve such a vision, it is critical to have a robust data and informatics infrastructure with the following properties: 1) high-throughput and real-time methods for data extraction and analysis, 2) transparent and reproducible processes to ensure scientific rigor in clinical research, and 3) implementable and generalizable scientific findings [1, 2, 4–7].

One common approach to practice-based knowledge discovery is chart review, a process of extracting information from patient medical records to answer a specific clinical question [8, 9]. Traditionally, chart review is performed manually and follows a pre-defined abstraction protocol [10]. Since a significant portion of clinical information is embedded in text, this manual approach can be time-consuming and costly [10–14]. With the implementation of EHRs, chart review can be automated by extracting data from structured fields systematically and leveraging natural language processing (NLP) techniques to extract information from text. Multiple studies have been reported to leverage NLP for extracting information from a diverse range of document types, such as clinical notes, radiology reports, and surgical operative reports [15–17], resulting in an effort reduction of up to 90% in chart review [18]. The development and evaluation of NLP algorithms for a specific chart review task requires the manual creation of a gold standard clinical corpus, however, there is a lack of standard processes or best practices for creating such a corpus [19, 20].

Meanwhile, reproducibility is crucial in the field of medicine where findings of a single site study must be able to be independently validated at different sites [21–24]. It is very challenging to validate an EHR-based study, due to the heterogeneity and complexity of EHR systems, the challenge of collaboration across diverse research stakeholders (i.e. physician, informatician, statistician, and IT), and the lack of standard processes and best practices for conducting EHR-based studies [19, 20, 25]. The lack of detailed study protocols, such as annotation guidelines and abstraction forms, can cause a study to not be reproducible, even at the same site [26]. For example, Gilbert et al. reviewed research articles published in three emergency medicine journals and discovered that among all studies related to retrospective chart review, only 11% reported the use of an abstraction form [14].

Challenges faced in leveraging EHR data lie in the voluminous, complex, and dynamic data being generated and maintained in heterogeneous sources. Madigan et al. systematically assessed the variability of 10 different clinical databases and discovered that between 20 to 40% of observational database studies can alter from statistically significant in one direction to an opposite direction [27]. A research study conducted by Sohn at el, assessing clinical documentation variations across two different EHRs, discovered potential corpus variability (i.e. number of clinical concepts per patient and number of clinical concepts per document are different) in unstructured text [28]. Another challenge found between heterogeneous EHRs is missing and noisy data [29]. Since different EHRs may have different causes underlying their missing data, unintentional bias may be introduced if the issue is ignored or poorly handled [30]. These variations and challenges need to be considered when developing solutions for information extraction and knowledge translation. To facilitate multi-site studies [4], efforts are underway to link EHRs across institutions and to standardize the definition of phenotypes for large-scale studies of disease onset and treatment outcomes in routine clinical care [31–34], however, unstructured data still remains a challenge.

In the clinical NLP community, efforts have been made to standardize corpus development including building and sharing annotated lexical resources, normalizing data elements, and developing an ontology-based web tool [13, 35–37]. However, to the best of our knowledge, there has been little informatics investigation done regarding the impact of using EHRs for clinical research in multi-institutional settings. Here, we conducted a multi-site EHR-based case study in the ESPRESSO (Effectiveness of Stroke Prevention in Silent Stroke) [38] project involving multiple steps to generate a corpus for the development of complex phenotype algorithms. The heterogeneity of healthcare institutions, EHR systems, documentation, and process variation in case identification was assessed quantitatively and qualitatively.

## Methods

### Data abstraction framework for EHR-based clinical research

**Fig. 1:**
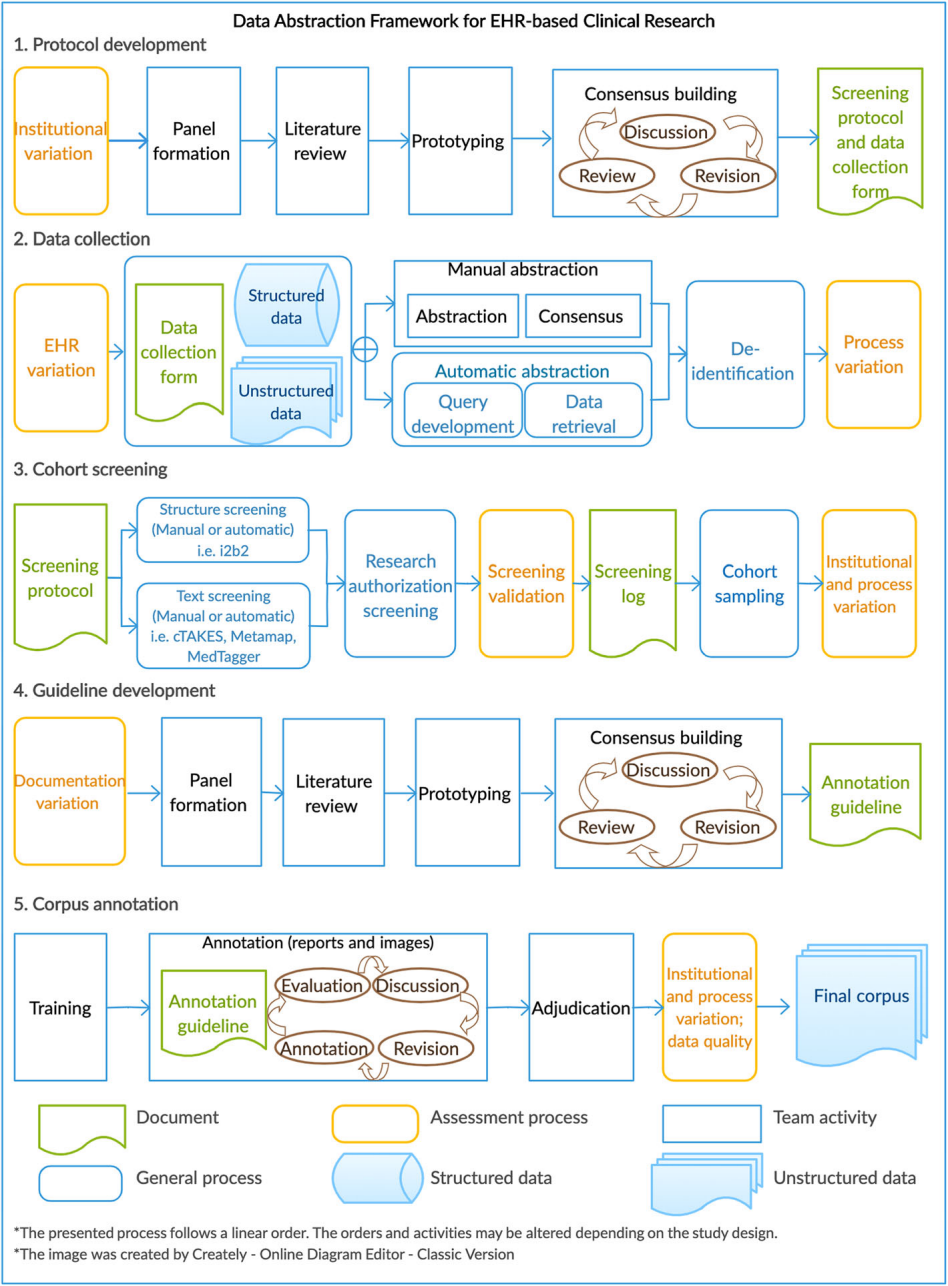
Data Abstraction Framework for EHR-based Clinical Research

We developed a data abstraction framework to standardize the process for multi-site EHR-based clinical studies aiming to enhance research reproducibility. The development of the proposed framework was designed after review of the existing guidelines and best practices, including *Corpus Annotation Schemes* [39]; *Fundamentals of clinical trials* [40]*;* and *Research data management* [41]*.* Figure 1 presents the process of creating annotated corpora for EHR-based clinical research.

The framework summarizes the linear process of extracting or reviewing information from EHRs and assembling a data set for various research needs. The processes consider important action items and documentation checklist to identify, evaluate and mitigate variations across sites. Depending on the study design, the order of processes and selection of activities can be altered. We considered four types of variations: institutional variation, EHR system variation, documentation variation, and process variation (Fig. 1, yellow boxes). Table 1 summarizes the definitions, potential implication and assessment methodologies of these variations.

**Table 1:** Variation Assessment Table for Data Abstraction

**Variation Type**	**Definition**	**Potential Implication**	**Example of Assessment Method**
Institutional variation	Variation in practice patterns, outcomes, and patient sociodemographic characteristics	Inconsistent phenotype definition; unbalanced concept distribution	• Compare clinical guideline, protocol, and definition • Calculate the number of eligible patients divided by screening population • Calculate the ratio of the proportion of the persons with the disease over the proportion with the exposure
EHR system variation	Variation in data type and format caused by different EHR system infrastructure	Inconsistent data type; different data collection processes	• Compare data type, document structure, and metadata • Conduct a semi-structured interview to obtain information about the context of use
Documentation variation	Variation in reporting schemes during the processes of generating clinical narratives	Noisy data	• Compare the cosine similarity between two documents represented by vectors • Conduct a sub-language analysis to assess syntactic variation
Process variation	Variation in data collection and corpus annotation process	Poor data reliability, validity, and reproducibility	• Calculate the degree of agreement among abstractors • Conduct a semi-structured interview to obtain information about the context of use

### A case study – the ESPRESSO study

This ESPRESSO study is an EHR-based study aiming to estimate the comparative effectiveness of preventive therapies on the risk of future stroke and dementia in patients with incidentally-discovered brain infraction [38, 42]. The study has been approved by the Mayo Clinic and Tufts Medical Center institutional review boards. Mayo Clinic is a tertiary care, nonprofit, academic medical center. Mayo Clinic is a referral center with major campuses in three regions of the U.S. including Rochester, Minnesota; Jacksonville, Florida; and Phoenix/Scottsdale, Arizona as well as Mayo Clinic Health System locations that serve more than 70 communities in Iowa, Wisconsin and Minnesota. The organization attends to nearly 1.2 million patients each year, who come from throughout the United States and abroad. The Saint Mary’s (1,265 licensed beds) and Rochester Methodist (794 beds) campuses are two main hospitals located in Rochester, Minnesota. Tufts Medical Center is similarly a tertiary care, nonprofit, academic medical center that is located in Boston, MA and is the principal teaching hospital of the Tufts University School of Medicine. The 415 licensed bed medical center provides comprehensive patient care across a wide variety of disciplines with disease-specific certifications through the Joint Commission as a Comprehensive Stroke Center and transplant center. TMC is the referral center for the WellForce network serving communities throughout Eastern Massachusetts and New England (Maine, New Hampshire, Vermont, Rhode Island). The medical center is actively engaged in clinical research and medical education with ACGME-accredited residencies and fellowships.

Silent brain infarction (SBI) is the presence of one or more brain infarcts, presumed to be due to vascular occlusion, found by neuroimaging in patients without clinical manifestations of stroke [43–45]. It is more common than a stroke and can be detected in 20% of healthy elderly people [43–45]. Early detection of SBI may prompt efforts to mitigate the risk of stroke by offering preventative treatment plans. In addition to SBI, white matter disease (WMD) or leukoaraiosis is another common finding in neuroimaging of older patients. SBI and WMD are related, but it is unclear whether they result from the same, independent, or synergistic processes [46, 47]. Since SBIs and WMDs are usually incidentally detected, there are no related International Classification of Diseases (ICD) codes in the structured fields of EHRs to facilitate large-scale screening. Instead, the findings are usually recorded in neuroimaging reports, so NLP techniques offer an opportunity to systematically identify SBI and WMD cases in EHRs.

In the study, we demonstrated the process of using EHRs for developing complex phenotypes to identify individuals with incidentally-discovered SBIs and WMDs. The process was assessed by corpus statistics, screening ratio, prevalence ratio, inter-annotator agreement, and qualitative interview.

### Methodologic process of using EHRs

#### Protocol development

A screening protocol was co-developed by the two institutions using procedure codes, diagnosis codes, and problem lists. The protocol included ICD-9 and ICD-10 codes to identify non-incidental clinical events. The codes were expanded with the corresponding descriptions to enable us to perform a text search. The full ICD-9 and ICD-10 codes and key terms are listed in the Additional file 1. The initial criteria were developed by a vascular neurologist at TMC and were evaluated by two neuroradiologists and one internist. The inclusion criteria were defined as individuals with neuroimaging scans between 2009 and October 2015. The exclusion criteria included patients with *clinically-evident stroke, transient ischemic attack (TIA)*, and *dementia* any time before or up to 30 days after the imaging exam. TIA was considered an exclusion criterion as TIA is sometimes incorrectly assigned on occasion by clinicians as the diagnosis in the setting of transient neurologic symptoms and positive evidence of brain infarction on neuroimaging. Dementia was an exclusion criterion because of a projected future application of the NLP algorithm in identifying patients for comparative effectiveness studies or clinical trials for which both stroke and dementia could be outcomes of interest. The systematic reviews suggested that the U.S. population over 50 years old had a high average prevalence of SBI [44]. By identifying a large cohort of patients with SBIs, age restriction was applied to exclude individuals 50 years of age or younger at the time of the first neuroimaging scan.

#### Data collection

At TMC, the data was aggregated and retrieved from three EHR systems: General Electric Logician, eClinicalWorks, and Cerner Soarian. The EHRs in TMC were implemented in 2009 with 1,031,159 unique patient records. At Mayo Clinic, the data was retrieved from the Mayo Unified Data Platform (UDP), an enterprise data warehouse which loads data directly from Mayo EHRs. Mayo EHR was implemented in 1994. Currently, there are 9,855,533 unique patient records. To allow data sharing across the sites, we de-identified the data by applying the de-identification tool DEID [48], a Java-based software that automatically removes protected health information (PHI) in neuroimaging reports with manual verification where an informatician, an abstractor and a statistician manually reviewed all the output from DEID.

#### Cohort screening

At Mayo Clinic, an NLP system, MedTagger [49], was utilized to capture mentions from the exclusion list in the clinical notes. As the system has a regular expression component, language variations such as spelling and abbreviations were able to be captured. Structured ICD-9 and ICD-10 codes were obtained by an informatician from the UDP. A clinician and an abstractor manually compared the screened cohort with the EHRs to ensure the validity of the screening algorithm.

At TMC, due to infrastructure limitations, this process was conducted through manual chart review. To ensure reproducibility, we carefully documented each step of the workflow (Additional file 3). Briefly, a vascular neurologist and three research assistants conducted manual chart review in order to determine whether individuals were included or excluded appropriately at each step. This process was performed using a list of free text exclusion criteria associated with the exclusionary ICD-9 and ICD-10 codes. It involved review of the full text of any discharge summaries associated with the encounter during which the neuroimaging scan was obtained in Cerner Soarian, if present, as well as review of the neuroimaging scan indication in the neuroimaging report.

Each site randomly selected 500 eligible reports to form the raw corpus for guideline development and corpus annotation. The cohort consisted of 1400 reports with 400 duplications for double reading. Among the total 400 double-read reports, 5 reports were removed because of invalid scan types. The remaining 395 reports were comprised of 207 from Mayo and 188 from TMC.

#### Guideline development

A baseline guideline was created by a vascular neurologist based on domain knowledge and published literature. To develop the annotation guideline, 40 reports pooled from the two institutions were annotated by two neuroradiologists and one neurologist using the baseline guideline. Inter-annotator agreement (IAA) was calculated and a consensus was organized to finalize the guideline, which included task definitions, annotation instructions, annotation concepts, and examples. The full guideline is provided in the Additional file 2.

#### Corpus annotation

**Fig. 2:**
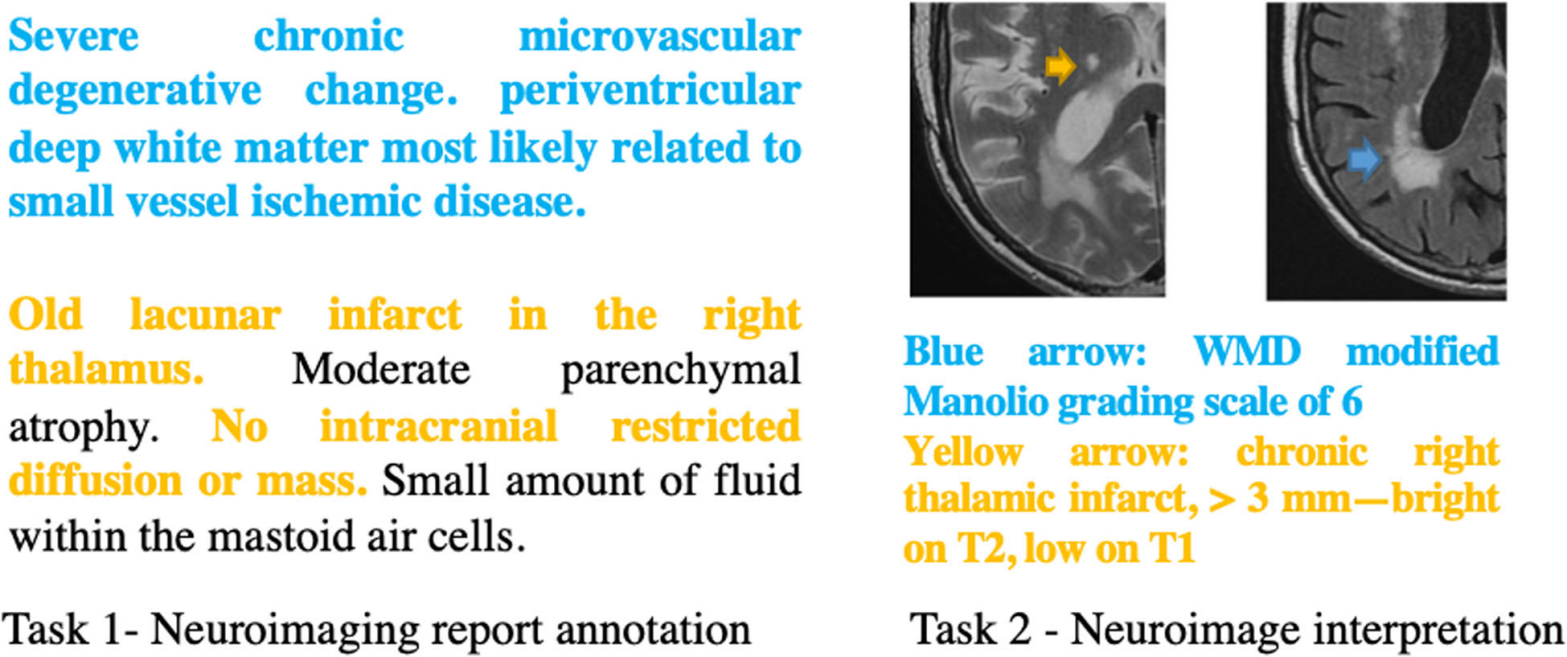
Example of neuroimaging report annotation (left) and neuroimage interpretation (right) for SBI (yellow) and WMD (blue)

The annotation processes consist of two tasks: neuroimaging report annotation and neuroimage interpretation. Neuroimaging report annotation is the process of reading and extracting SBI and WMD related sentences or concepts from text documents. Neuroimage interpretation is the process of identifying SBIs or WMDs from CT or MRI images. Figure 2 provides an example of two tasks.

##### Neuroimaging report annotation

The purpose of the annotation task was to annotate the findings of SBI and WMD lesions in both the body (Findings) and summary (Impression and Assessment) sections of neuroimaging reports. The annotation was organized into two iterations. The first iteration extended from the finalization of the process guideline until the midpoint when half of the reports were annotated. The goal of the first iteration was to identify new problems that were not captured in the sample data. After the first iteration, all problematic cases were reviewed by the two senior clinicians, and the guidelines were updated. The second iteration of annotation then commenced using the updated guidelines. Several consensus meetings were organized to resolve all disagreements after the annotation process was completed. All conflicting cases were adjudicated by the two senior clinicians. All of the issues encountered during the process were documented.

The annotation team was formed with members from both institutions. Two third-year residents from Mayo and two first-year residents from TMC performed the annotation. The experts for quality control were two senior radiologists from Mayo and one senior internist and one vascular neurologist from TMC. We used Multi-document Annotation Environment (MAE) [50], a Java-based natural language annotation software package, to conduct the annotation.

Prior to annotation, training was conducted for all four annotators including one online video session and two on-site training sessions. The online video provided demonstrations and instructions on how to download, install, and use the annotation software. The on-site training conducted by two neuroradiologists contained initial annotation guideline walkthroughs, case studies, and practice annotations. The same clinicians supervised the subsequent annotation process.

##### Neuroimage interpretation

To assess the validity of the corpus, we obtained a balanced sample of images with and without SBI from the annotated neuroimaging reports. From each site, 81 neuroimages were de-identified and reformatted to remove institution-specific information and then pooled together for the sample group. We invited four attending neuroradiologists, two from each site, to read grade the imaging exams. Each exam was graded twice by two neuroradiologists independently. The image reading process followed the proposed best practices including guideline development, image extraction form, training, and consensus building. The level of agreements between the research grade reading of the neuroimages and the corresponding annotation of the reports was calculated.

#### Assessment of heterogeneity

The screening ratio was calculated on the post screened cohort. Cohen’s kappa [51] and F-measure [52] were adopted to measure the IAA during the annotation and image reading processes. Corpus statistics were used to measure the variations in clinical documentation across institutions. The analysis compared corpus length, number of SBI and WMD concepts, number of documents with SBI and WMD concepts, and distribution of SBI related concept mentions. Document similarity was calculated by comparing the cosine similarity between two vectors created by term frequency-inverse document frequency (tf-idf), where each corpus was represented by a normalized tf-idf vector [28]. Age-specific prevalence of SBI and WMD were calculated and compared with the literature. To analyze the cohort characteristics between Mayo and TMC, Student’s t-test was performed for continuous variables. Comparison of categorical variables was calculated with frequency tables with Fisher’s exact test.

Qualitative assessments were conducted to evaluate the abstraction process and an assessment protocol was created to facilitate the post abstraction interview. The protocol was designed to focus on three main areas: 1) evaluation of the abstraction process, 2) language patterns in the reports, and 3) abstraction techniques. Four back-to-back interviews were conducted with the four abstractors following the guidelines of Contextual Interview (CI) suggested by Rapid Contextual Design [53]. Each interview was conducted by an informatician and lasted approximately 30 min. Questions and issues raised by each annotator during the two iterations of annotation were collected and qualitatively assessed. The data were then classified into six categories: data, modifier, medical concept, annotation rules, linguistic, and other.

## Results

### Corpus annotation

#### Neuroimaging report annotation

The average inter-annotator agreements across 207 Mayo reports and 188 TMC reports on SBI and WMD were 0.87 and 0.98 in kappa score and 0.98 and 0.99 in F-measure, respectively. Overall, both Mayo and TMC annotators achieved high inter-annotator agreements.

#### Neuroimage interpretation

The average inter-annotator agreement among four neuroradiologists was 0.66 in kappa score and 0.83 in F-measure. The average agreement between neuroimaging interpretation and corpus annotation was 0.68 in kappa score and 0.84 in F-measure. The result suggested high corpus validity outcomes.

### Assessment of heterogeneity

#### Institutional variation

The process of screening eligible neuroimaging reports across two institutions was variant. At Mayo, 262,061 reports were obtained from Mayo EHR based on the CPT inclusion criteria. 4015 reports were randomly sampled for cohort screening. 749 were eligible for annotation after applying the ICD exclusion criteria (structured and unstructured). At TMC, 63,419 reports were obtained from TMC EHR based on CPT inclusion criteria. 12,092 reports remained after applying the ICD exclusion criteria (structured). 1000 reports were randomly selected for text screening, a method of identifying eligible patients using NLP techniques to extract eligibility criteria from patient clinical notes. 773 reports were eligible for annotation. Among the total 1522 eligible (Mayo 749, TMC 773) neuroimaging reports, 1000 (Mayo 500, TMC 500) reports were randomly selected.

The prevalence of SBI and WMD for Mayo and TMC patients at age of 50, 60, 70 and 80 is listed in Table 2. Despite the variation, the results were consistent with the published literature, between 10 and 20% [43–45], and the number increased with age in both computed tomography (CT) and magnetic resonance imaging (MRI) as anticipated.

**Table 2:** The prevalence of SBI and WMD for Mayo and TMC patients at age of 50, 60, 70 and 80

	**CT Scan (%) - SBI**		**MRI Scan (%) - SBI**		**CT Scan (%) - WMD**		**MRI Scan (%) - WMD**	
**Age**	**Mayo**	**TMC**	**Mayo**	**TMC**	**Mayo**	**TMC**	**Mayo**	**TMC**
> = 50	12.5	7.4	11.3	7.7	28.7	55.0	69.2	51.7
> = 60	16.0	9.4	14.0	9.7	35.1	65.9	75.3	60.2
> = 70	23.5	11.4	20.2	12.2	47.1	80.7	84.6	65.3
> = 80	26.3	18.4	26.5	20.8	52.6	94.7	85.3	66.7

The average age of Mayo and TMC patients 65 and 66, respectively. The number of female patients in the Mayo and TMC cohort were 243 and 274, respectively. We found a moderate variation in the presence of SBI and WMD and a high variation in the WMD grading. A significant variation in the missing documentation of WMD grading between Mayo and TMC was found (*p* = 0.0024). Table 3 summarizes the cohort characteristics across two institutions.

**Table 3:** Analysis of Cohort Characteristics Between Mayo and TMC

**Variables**	**Mayo (*n*** **= 500)**	**TMC (*n*** **= 500)**	***p*** **Value**
Age (mean)	65 (+ − 10.6)	66 (+ − 9.7)	0.1197
Gender (female)	243 (48.6)	274 (54.8)	0.0576
SBI	57 (11.4)	38 (7.6)	0.0516
Acuity			
Acuity/subacute	6 (1.2)	6 (1.2)	1.0000
Chronic	44 (8.8)	29 (5.8)	0.0882
Non-specified	7 (1.4)	3 (0.6)	0.3407
Location			
Lacunar/subcortical	27 (5.4)	10 (2.0)	**0.0065**
Cortical/juxtacortical	9 (1.8)	13 (2.6)	0.5188
Both	0 (0)	3 (0.6)	0.2492
Non-specified	21(4.2)	12 (2.4)	0.1558
WMD	291 (58.2)	264 (52.8)	0.9800
WMD grading			
Mild	191 (38.2)	154 (30.8)	**0.0165**
Mild/moderate	21 (4.2)	0 (0.0)	**7.6963e-7**
Moderate	42 (8.4)	45 (9.0)	0.8226
Moderate/severe	2 (0.4)	0 (0)	0.4995
Severe	8 (1.6)	11 (2.2)	0.6443
No mention of quantification	27 (5.4)	54 (10.8)	**0.0024**

*Definition of abbreviations: CI confidence interval, OR odds ratio*

#### EHR system variation

There was a high variation in the EHR system vendors, the number of EHR systems per site, and the extract, transform, and load (ETL) processes for the different EHR systems between Mayo and TMC. At TMC, the data was obtained directly from three EHR systems: General Electric Logician, eClinicalWorks, and Cerner Soarian. The data retrieval process involved difference abstraction processes due to the different interface design and data transfer capabilities. At Mayo Clinic, there was an ETL process to aggregate the data from Mayo EHRs to the enterprise data warehouse. Since data could be linked and transferred through direct queries, the abstraction process was less variant.

#### Documentation variation

There was variation between Mayo and TMC in expressing SBI and WMD in neuroimaging reports. Corpus statistics identified the three most frequent expressions of negated infarction in neuroimaging reports (Table 4). In the TMC reports, “no acute territorial infarction” is a common phrase to describe negated SBI concepts. This expression was never discovered in Mayo reports. When describing the grade measure for WMDs, definitive expressions such as “mild”, “moderate” and “severe” were used by Mayo physicians. On the other hand, TMC physicians used more descriptive expressions in describing the grade measure for WMDs. In regards to documentation styles, TMC used a template-based reporting method whereas Mayo did not adopt any reporting schemas. The average numbers of tokens per document on Mayo and TMC reports were 217 and 368, respectively. The corpus similarity between TMC and Mayo Clinic radiology reports was 0.82 and suggested a potential moderate-to-high semantic similarity. Overall, Mayo’s reports are definitive and varied, whereas TMC reports are lengthy, standardized and descriptive.

**Table 4:** Example of Language Variation between Two Data Sources

**Mayo – Non-SBI**	**TMC – Non-SBI**
• No restricted diffusion. • No focal masses, focal atrophy, or foci of restricted water diffusion. • No evidence for acute ischemia on the diffusion weighted images.	• There is no acute territorial infarct. • No acute territorial infarct. • There is no decreased diffusion to indicate an acute infarct.
**Mayo – WMD**	**TMC – WMD**
• Mild leukoaraiosis • Minimal leukoaraiosis • Moderate leukoaraiosis	• There are scattered foci of hypodensity in the subcortical and periventricular white matter, a non-specific finding but likely reflecting the sequela of chronic microangiopathy • Areas of white matter hypodensity are a non-specific finding but may represent the sequela of chronic microangiopathy • There are multiple foci of t2 flair hyperintensity in the periventricular, deep and subcortical white matter, a non-specific finding but likely reflecting the sequela of chronic microangiopathy

#### Process variation

**Fig. 3:**
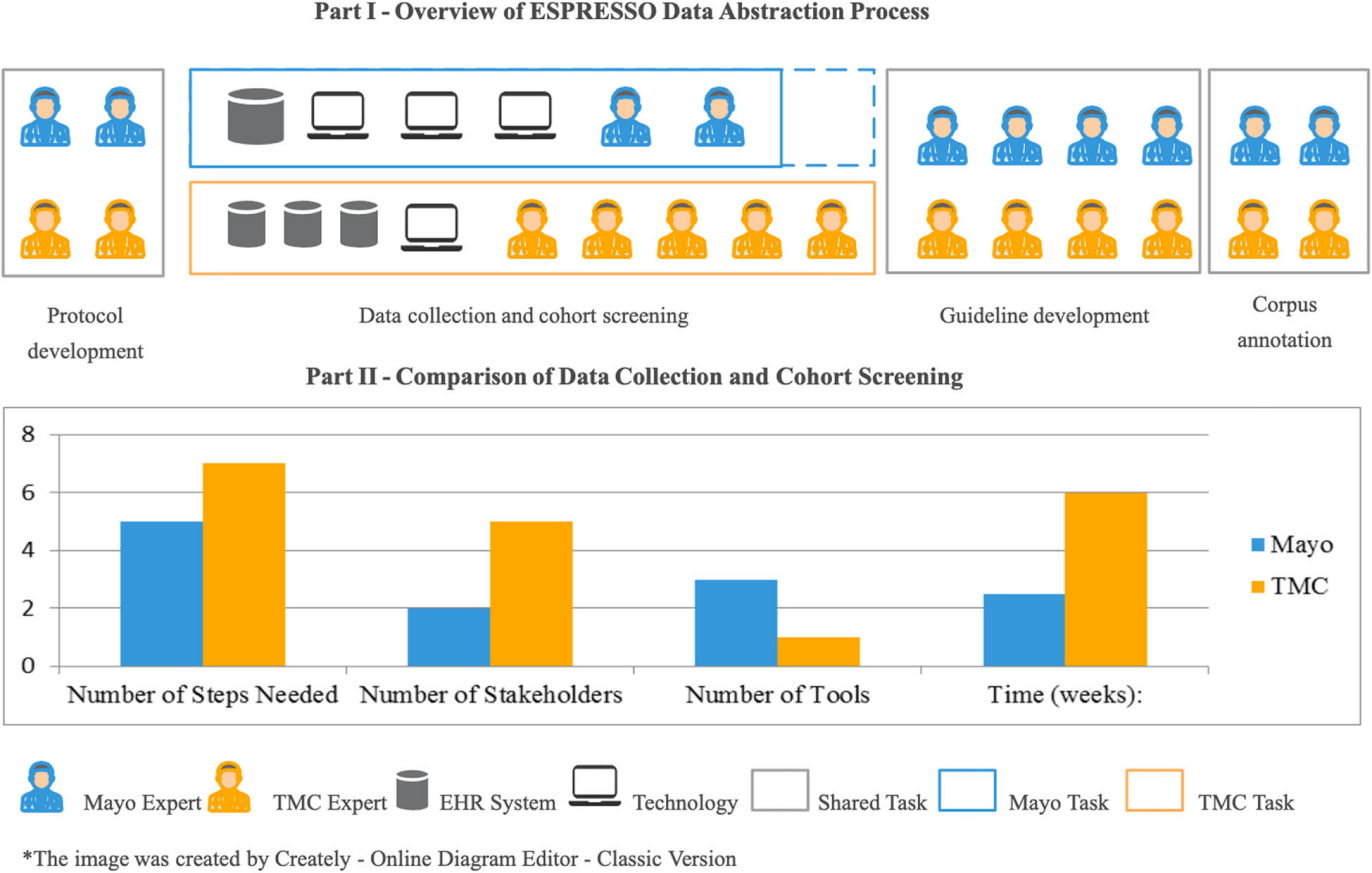
Overview of ESPRESSO Data Abstraction Process. Total Annotation Issues during Two Iterations

The process map of the ESPRESSO data abstraction is illustrated in Fig. 3 – Part I. The map provides an overview of the relationship and interaction between people and technology in the context of the data abstraction process. The analysis suggested that the variations of EHR systems and technology infrastructures between the two sites have resulted in differences in the number of processing steps, experts, and duration (Fig. 3 – Part II).

### Recommended practices for EHR-based data abstraction

Throughout this case study, we encountered many challenges during the data abstraction process. Here we summarize some lessons learned and provide a few empirical recommendations to promote the best practices of using EHRs for clinical research.

#### Institutional variation

It is inevitable to encounter variabilities across different institutions. Being aware of the degree of variation can help estimate biases and prevent inaccurate study conclusions. Thus, it is always helpful to apply informatics techniques to capture and assess the variation to ensure transparent and informed EHR-based clinical research.

#### Documentation plan and checklist

A comprehensive documentation plan for a study allows interventions aimed at process replication and error prevention to be designed into the data abstraction. The plan should explicitly mention what, where, and when to document experimental elements such as protocols, guidelines, codes, operations manuals, and process workflows. Ensuring adequate time is devoted to documentation is critical in order to prevent details from being overlooked or omitted. A documentation checklist ensures important study details are documented. Examples of important metadata elements are data identifier (i.e. document id, document date, and patient clinic number), cohort definition (i.e. inclusion and exclusion criteria), steward, and description of the data (when the data is created, moved, modified, or filtered). During data abstraction, process logs, tools, data definitions, and methodologies need to be carefully analyzed and explicitly stated.

#### Concept definitions and protocol co-development

To ensure data validity, the variables of the study should be strictly defined. Standardized terminology codes, such as ICD, SNOMED-CT, CPT-4, or RxNorm are useful for describing observable medical characteristics. Protocol co-development and consensus building helped reduce institutional and process variance in our study (Figs. 1 and 3). Particularly, having a well-represented expert panel (from all sites) for developing and evaluating inclusion and exclusion criteria and annotation guidelines helped the creation of high-quality protocol documents.

#### Abstraction and annotation training

Proper training and education can help reduce process inconsistency and increase transparency, especially for a cross disciplinary team. When the training sessions were applied, a shared understanding of rigorous experimental design, research standards, and objective evaluation of data was ensured. Some example training activities included discussing the overall study goal, going through the contents of the annotation guideline and definitions of interest, and practicing using the annotation tool (i.e. allowing people to work on a sample of 5–10 notes).

#### Process iteration and consensus building

A consensus reaching process is an iterative and dynamic process for building agreement on any potential issues and disagreements. A consensus meeting should be organized when developing screening protocols and annotation guidelines. Routine discussions ensure guidelines and protocols are scientifically valid and robust.

#### Adoption of appropriate informatics tools

Successfully leveraging informatics techniques can improve process efficiency, data quality, and reproducibility. For example, automatic data retrieval techniques (such as application programming interface and structured query language) and cohort screening tools (such as i2b2 [54]) can enable a high-throughput data abstraction process. Using annotation tools ensures a standardized and reproducible annotation process. It is more important to choose an appropriate informatics solution than an advanced solution. In the study, we chose a light and standalone version of annotation software over an advanced web-based tool due to its high feasibility and efficiency. In situations that require extensive validation for processes, such as de-identification, human validations are needed after applying the informatics tools.

## Discussion

We conducted a multi-site EHR-based case study in the implementation of the ESPRESSO project to assess the impact of EHR heterogeneity for clinical research. The case study discovered significant variation regarding patient population, neuroimaging reporting, EHR systems, and abstraction processes. Despite the variation, the evaluation of the final corpus yielded high reliability and validity.

The assessment through the ESPRESSO discovered a high variation in the reported prevalence of SBIs between Mayo and TMC. There are two potential reasons for the low prevalence of SBIs in TMC. First, the two locations have different patient sociodemographic characteristics at the two locations. Although both Mayo and TMC are referral centers, Mayo may have a larger proportion of patients who are referred from distant locations whereas TMC may have predominantly local and regional referrals. Second, low SBI prevalence may be due to the different documentation priorities during the routine practice. For further investigation, a qualitative assessment was utilized to learn how clinicians report neuroimaging interpretations. Based on the analysis of cohort characteristics between Mayo and TMC (Table 3) and the post abstraction interview, we discovered a portion of SBIs were under-documented by TMC neuroradiologists due to their historical perceptions of potentially low clinical significance for SBIs. For example, the descriptions about the clinical utility of reporting on small and presumably asymptomatic brain lesions that could represent infarcts were very uncertain. Compared with TMC, the wording describing SBIs on the Mayo reports was more definitive.

Although the average kappa score on the Mayo reports was lower than the TMC reports, the score still reflected an exceptionally high agreement between all annotators. We believe this was achieved by a well-designed process. During guideline development, we found that variation could be reduced by adding an instruction manual to the guidelines. Due to the large number of reports that were assigned to each resident, the de-identified reports were equally distributed to individuals as a “take home” assignment. The instruction manual helped to guide annotation activities, such as suggesting the number of reports that needed to be annotated per day. One of the most commonly raised issues was the lack of precise modifier definitions for WMD. To reduce the abstraction variation caused by different interpretation of modifiers, we created a normalization mapping schema. For example, the level of grading for WMDs was explicitly defined to be mild, mild/moderate, moderate, moderate/severe, and severe.

The qualitative assessment of the annotation process (Fig. 1 - process 5 - box 2) identified that medical concepts (i.e. mention of SBI and WMD) and modifiers (i.e. acuity and location) were the primary issues during the first iteration of annotation. Additional training was offered to address the primary issues experienced during the first iteration of annotation and thus, decreased the occurrence of issues during the second iteration (Fig. 3). All four annotators noted that with the combination of training and comprehensive annotation guidelines, annotation time was shortened, effort redundancy was reduced, and annotation consistency was improved.

## Limitations and future work

Since the study was conducted on two sites with one case scenario, the generalizability of the process is limited by the scope of the study. Our next step is to expand our investigation on pragmatic clinical trials by incorporating more sites and case scenarios. Furthermore, we plan to develop a standardized process framework for EHR-based clinical research to ensure the validity, reliability, reproducibility and transparency of research findings.

## Conclusion

We conducted a case study based on the ESPRESSO project identified the institutional and process variations and the heterogeneity of EHRs across sites. Our experiment demonstrates the necessity to have a standardized process for the use of EHRs for clinical studies.

## Supplementary information

**Additional file 1:** Supplemental Appendix 1. Screening Protocol.

**Additional file 2:** Supplemental Appendix 2. Annotation Guideline.

**Additional file 3:** Supplemental Appendix 3. Process Documentation TMC Manual Screening.

## Data Availability

The datasets used and/or analyzed during the current study are available from the corresponding author on reasonable request.

## Abbreviations

EHR: Electronic Health Records
TMC: Tufts Medical Center
LHS: Learning Health System
NLP: Natural Language Processing
ESPRESSO: Effectiveness of Stroke Prevention in Silent Stroke
WMD: White Matter Disease
SBI: Silent Brain Infraction
ICD: International Classification of Diseases
PHI: Protected Health Information
IAA: Inter-annotator Agreement
MAE: Multi-document Annotation Environment
TF-IDF: Term Frequency-Inverse Document Frequency
MRI: Magnetic Resonance Imaging
CT: Computed Tomography
ETL: Extract, transform, and load
