# Supplementary material for: Assessment of the impact of EHR heterogeneity for clinical research through a case study of silent brain infarction
Source: BMC Med Inform Decis Mak. 2020 Mar 30;20:60. doi: 10.1186/s12911-020-1072-9 (PMC7106829; doi:10.1186/s12911-020-1072-9)
Supplement: Supplementary file 1 — Additional file 1: Supplemental Appendix 1. Screening Protocol. [file 12911_2020_1072_MOESM1_ESM.docx]

**SUPPLEMENTAL** **APPENDIX 1. Screening Protocol**

**Instructions:**the screening protocol consists of two parts: ICD screening and text screening. For ICD screening (Step 1-3), all steps can be performed using cohort screening and feasibility assessment tools. In our study, we used i2b2 (<https://www.i2b2.org/>) to perform the ICD screening. If the institution does not have a screening tool, this step can be performed through custom SQL or statistical software such as SAS or R. The text screening (step 4) can be performed using existing NLP frameworks, such as cTAKES (<https://ctakes.apache.org/>

). In our study, we used MedTagger (<https://github.com/OHNLP/MedTagger>) to perform text screening. The tool has a regular expression component, and language variations such as spelling and abbreviations were able to be captured.

Step 1: apply ICD codes in part 1 – I to obtain Cohort A

Step 2: apply ICD codes in part 1 – II on Cohort A to obtain Cohort B

Step 3: remove Cohort B from Cohort A. This step produces Cohort C

Step 4: apply keywords in part 2 on Cohort C to obtain Cohort D

Step 5: remove Cohort D from Cohort C. This step produces the final cohort

# Part 1 - ICD9/10 Screening (Inclusion and Exclusion)

1. Inclusion Criteria – applied at the time of the index scan
   1. The patient has any of the following **neuroimaging scans** (CPT codes, or cross walked to ICD9/10 codes):
      1. **Head CT** 70450 (noncontrast), 70460 (with contrast), 70470 (with and without contrast)
      2. **Brain MRI** 70551 (noncontrast), 70552 (with contrast), 70553 (with and without contrast)
   2. Clinical encounter documentation describing the **indication** or **context** for the scan (at least one of the following):
      1. **Outpatient evaluation** (primary care or subspecialty)
         1. Clinic note
      2. **Inpatient evaluation**
         1. Discharge summary

*Note: Emergency department evaluations without admission to the hospital are excluded as they [1] may not be sufficiently comprehensive in acquiring past medical history data, and [2] do not routinely have the opportunity to provide continuity of care (i.e. response to imaging findings) if the final Radiology interpretation occurs after the completion of the clinical encounter. This also includes ED visits with subspecialty consultations (i.e. Neurology) where the patient is not hospitalized following the ED visit.

**Based on discussions with the TMC Informatics team, we have simplified the manual screening process to only include discharge summaries (for inpatients).

1. Exclusion criteria – applied at the time of the index scan
   1. Informatics and/or manual EMR screen should review the following fields:
      - 1. Informatics = structured data fields (inpatient or outpatient)
           1. **Problem list** (from any clinical encounter document associated with the ordering of the scan or within 30 days after the scan)
           2. **Billed diagnoses** (from any clinical encounter document associated with the scan or within 30 days after the scan)
        2. Manual = discharge summary (inpatient only)
           1. **Principal/discharge diagnosis** (from discharge summary within 30 days after the scan)
           2. **Past medical history** (from discharge summary within 30 days after the scan)
           3. **Hospital course** (from discharge summary within 30 days after the scan)
   2. **Outcomes - Prior** history or **new** diagnosis of clinically-apparent **ischemic stroke**, **TIA**, **stroke symptom (high likelihood)**, or **dementia**
      1. ICD9 exclusions
         1. Ischemic stroke or TIA (of the brain, retina, or spinal cord)
            1. **434.0 series - cerebral thrombosis**

434.00 cerebral thrombosis without cerebral infarction

434.01 cerebral thrombosis with cerebral infarction

- - - - 1. **434.1 series - cerebral embolism**

434.10 cerebral embolism without cerebral infarction

434.11 cerebral embolism with cerebral infarction

- - - - 1. 433 occlusion and stenosis of precerebral arteries (only selecting codes with cerebral infarction as other codes may represent asymptomatic stenosis/occlusion)

**433.01 occlusion and stenosis of basilar artery with cerebral infarction**

**433.11 occlusion and stenosis of carotid artery with cerebral infarction**

**433.21 occlusion and stenosis of vertebral artery with cerebral infarction**

**433.31 occlusion and stenosis of multiple and bilateral precerebral arteries with cerebral infarction**

**433.81 occlusion and stenosis of other specified precerebral artery with cerebral infarction**

**433.91 occlusion and stenosis of unspecified precerebral artery with cerebral infarction**

- - - - 1. **435 series** - transient cerebral ischemic

435.8 transient cerebral ischemic NEC

435.9 transient cerebral ischemia NOS

- - - - 1. **V12.54** personal history of transient ischemic attack, and cerebral infarction without residual deficits
        2. **997.02** iatrogenic cerebrovascular infarction or hemorrhage
        3. **362 series** - retinal artery occlusion

362.30 retinal vascular occlusion NOS

362.31 central retinal artery occlusion

362.33 partial arterial occlusion

362.32 arterial branch occlusion

362.34 transient arterial occlusion

- - - - 1. spinal cord infarct (no ICD9 codes)
      1. Stroke symptom (high likelihood) = stroke is likely to be the highest diagnosis on the differential diagnosis when ordering the head imaging study
         1. Late effects of cerebrovascular disease (cognitive, speech and language, hemiplegia/hemiparesis, monoplegia, paralytic syndrome, altered sensation, vision, apraxia, dysphagia, facial weakness, ataxia, vertigo)

**438 series**

- - - - 1. Aphasia

**784.3** aphasia

438.11 aphasia, late effect of cerebrovascular disease

**315.31** expressive language disorder

- - - - 1. Hemiparesis

Hemiplegia and hemiparesis

**342** hemiplegia and hemiparesis

**342.9** hemiplegia, unspecified

**344.9** paralysis NOS

Hemiplegia and hemiparesis, late effect

438.2 hemiplegia/hemiparesis, late effect of cerebrovascular disease

438.21 hemiplegia/hemiparesis, late effect, dominant side

438.22 hemiplegia/hemiparesis, late effect, non-dominant side

438.5 other paralytic syndrome, late effect of cerebrovascular disease

Facial weakness

438.83 facial weakness

**781.94** facial weakness

Limb weakness

**781.4** transient limb paralysis

**344.5** monoplegia NOS

Arm weakness

**344.4** monoplegia of upper limb

**344.40** monoplegia of upper limb unspecified side

**344.41** monoplegia of upper limb, dominant side

**344.42** monoplegia of upper limb, nondominant side

Leg weakness

**344.3** monoplegia of lower limb

**344.30** monoplegia of lower limb, unspecified side

**344.31** monoplegia of lower limb, dominant side

**344.32** monoplegia of lower limb, nondominant side

- - - - 1. Hemisensory loss (no ICD codes for acute sensory changes)

438.6 late effects

- - - - 1. Hemiataxia

438.84 ataxia late effect

**781.3** lack of coordination

**781.2** abnormality of gait

- - - - 1. Hemineglect

**781.8** neurologic neglect syndrome

- - - - 1. Visual disturbance

**368.40** visual field defect, NOS

**368.44** visual field defect, NEC

**368.84** visual field defects

**368.46** homonymous hemianopsia

**368.47** heteronymous hemianopsia

**368** visual disturbances

**368.8** visual disturbances NEC

**368.9** visual disturbance NOS

**369.8** visual loss, one eye nos

**369.9** visual loss NOS

**368.11** sudden visual loss

**368.12** transient visual loss

**950**.3 injury to visual cortex

**377.72** visual cortex disorder with vascular disease

**377.62** visual pathway disorder with vascular disease

**950.2** injury to optic pathways

**950.9** injury to optic nerve/pathways NOS

**799.53** visuospatial deficit

- - - - 1. Dysarthria

**784.51** dysarthria

438.12 dysarthria, late effects of cerebrovascular disease

- - - - 1. Dysphagia

**787.2** dysphagia

787.20 dysphagia NOS

787.21 dysphagia, oral phase

787.22 dysphagia oropharyngeal

787.23 dysphagia, pharyngeal

787.23 dysphagia, pharyngoesophageal

787.29 dysphagia NEC

438.82 late effects of cerebrovascular disease, dysphagia

- - - 1. Dementia (possibly related to cerebrovascular disease)
         1. **290** **series** (dementia)

290.0 senile dementia uncomplicated

290.10 presenile dementia uncomplicated

290.11 presenile dementia with delirium

290.12 presenile dementia with delusional features

290.13 presenile dementia with depressive features

290.20 senile dementia with delusional features

290.21 senile dementia with depressive features; with behavioral disturbance

290.30 senile dementia with delirium

290.40 vascular dementia, uncomplicated

290.41 vascular dementia, with delirium

290.42 vascular dementia, with delusions

290.43 vascular dementia, with depressed mood

- - - - 1. **294 series** persistent mental disorders due to conditions classified elsewhere (only selecting dementias)

294.1 dementia in conditions classified elsewhere

294.10 dementia, without behavioral disturbance

294.11 dementia, with behavioral disturbance

294.20 dementia, unspecified

294.20 dementia, unspecified without behavioral disturbance

294.21 dementia, unspecified with behavioral disturbance

- 1. **Conditions impeding assessment of treatment effect (*prior history or current diagnosis*)**
     1. Stroke, unspecified
        1. **436 acute but ill-defined cerebrovascular accident**
     2. Intracranial hemorrhage
        1. Mixed
           1. **800.2** closed with SAH, SDH, and EDH
           2. **800.7** open with SAH, SDH, and EDH
           3. **801.7** open with SAH, SDH, and EDH
           4. **801.2** closed with SAH, SDH, and EDH
           5. **803.2** closed with SAH, SDH, and EDH
           6. **803.7** open with SAH, SDH, and EDH
           7. **804.2** closed with SAH, SDH, and EDH
           8. **804.7** open with SAH, SDH, and EDH
        2. Traumatic intracranial hemorrhage
           1. **852 series** – SAH, SDH, or EDH following injury

852.0 SAH following injury without mention of open intracranial wound

852.00 traumatic SAH

852.01 SAH no coma

852.02 SAH brief coma

852.03 SAH mod coma

852.04 SAH prolonged coma

852.05 SAH deep coma

852.06 SAH coma NOS

852.09 SAH with concussion

852.1 SAH following injury with open intracranial wound

852.19 open SAH with concussion

852.2 SDH following injury without mention of open intracranial wound

852.20 traumatic SDH

852.21 SDH without coma

852.22 SDH brief coma

852.23 SDH mod coma

852.24 SDH prolonged coma

852.23 SDH deep coma

852.26 SDH coma NOS

852.29 SDH with concussion

852.3 SDH following injury with open intracranial

852.39 open SDH with concussion

852.4 extradural hemorrhage following injury without mention of open intracranial wound

852.40 traumatic EDH

852.42 EDH brief coma

852.43 EDH mod coma

852.44 EDH prolonged coma

852.45 EDH deep coma

852.46 EDH coma NOS

852.49 EDH with concussion

852.41 EDH no coma

825.5 extradural hemorrhage following injury with open intracranial wound

852.59 EDH with concussion

- - - - 1. **853 series** – other and unspecified intracranial hemorrhage following injury
      1. Nontraumatic intracranial hemorrhage
         1. **430 series** – subarachnoid hemorrhage
         2. **431 series** – intracerebral hemorrhage
         3. **432 series** - nontraumatic extradural hemorrhage

*432.0*

*432.1*

*432.9*

- - - 1. Intraventricular hemorrhage (no ICD9 codes specific for adult IVH)
    1. Traumatic brain injury
       1. **851 series** – cerebral laceration and contusion
       2. **854 series** – intracranial injury of other and unspecified nature
       3. **V15.52** – history of traumatic brain injury
    2. Other symptomatic intracranial pathologies
       1. Anoxic brain injury
          1. **348.1** anoxic brain damage
       2. Brain tumor
          1. **191 series** - malignant neoplasm of brain
          2. **239.6** – brain neoplasm NOS
       3. Brain abscess and other intracranial infections (excluding meningitis which is not expected to leave lasting deficits resembling stroke)
          1. **006.5** amebic brain abscess
          2. **013.3 series** - tuberculous abscess of brain

*013.30*

*013.31*

*013.32*

*013.33*

*013.34*

*013.35*

*013.36*

- - - - 1. **324.0 series -** intracranial abscess
        2. **326 series** – late effect, CNS abscess
      1. Multiple sclerosis
         1. **340 series** – multiple sclerosis
    1. Other forms of dementia
       1. **291.2** alcohol persistent dementia
       2. **292.82** drug persistent dementia
       3. **331 series** (other cerebral degenerations)
          1. 331.0 Alzheimer’s disease
          2. 331.1 frontotemporal dementia

*331.11*

*331.19*

- - - - 1. 331.2 senile degeneration of brain
        2. 331.3 communicating hydrocephalus
        3. 331.4 obstructive hydrocephalus
        4. 331.5 normal pressure hydrocephalus
        5. 331.6 corticobasal degeneration
        6. 331.8 other cerebral degeneration

331.82 dementia with Lewy bodies

332.83 mild cognitive impairment

- - 1. **ICD10 exclusions**
       1. **Map from ICD9 codes starting in October 2015**

*This list of exclusions does not include meningitis, intracranial foreign bodies, cranial neuropathies, and spinal cord disorders.

# Part 2 - Text Screening (Exclusion)

**Key terms to identify stroke, TIA, and dementia from EMR clinical documentation**

1. Outcomes of interest
   1. Stroke
      1. Stroke
      2. Acute stroke
      3. Chronic stroke
      4. Prior stroke
      5. Previous stroke
      6. Old stroke
      7. Ischemic stroke
      8. Acute ischemic stroke
      9. AIS
      10. Chronic ischemic stroke
      11. Cerebral infarction
      12. Acute cerebral infarction
      13. Chronic cerebral infarction
      14. Brain infarct
      15. Acute brain infarct
      16. Chronic brain infarct
      17. Cerebral embolism
      18. Cerebral thrombosis
      19. Retinal artery occlusion
      20. Retinal artery embolism
      21. Retinal artery thrombosis
      22. Central retinal artery occlusion
      23. Central retinal artery embolism
      24. Central retinal artery thrombosis
      25. Branch retinal artery occlusion
      26. Branch retinal artery embolism
      27. Branch retinal artery thrombosis
      28. CRAO
      29. BRAO
      30. Spinal cord stroke
      31. Spinal cord infarct
      32. Spinal cord infarction
   2. Transient ischemic attack
      1. Transient ischemic attack
      2. TIA
   3. Stroke symptoms (high likelihood that stroke is the highest item on the differential for head imaging with these symptoms) in HPI or Assessment & Plan (not Problem List or Past Medical History)
      1. Aphasia
      2. Hemiplegia
      3. Hemiparesis
      4. Arm weakness
      5. Leg weakness
      6. Hemisensory loss
      7. Face numbness
      8. Arm numbness
      9. Leg numbness
      10. Hemiataxia
      11. Arm ataxia
      12. Leg ataxia
      13. Gait ataxia
      14. Hemineglect
      15. Visual field defect
      16. Quadrantanopia
      17. Hemianopia
      18. Amaurosis fugax
      19. Monocular vision loss
      20. Diplopia
      21. Dysarthria
      22. Dysphagia
      23. Apraxia
      24. Vertigo
   4. Dementia (related to cerebrovascular disease)
      1. Dementia
      2. Senile dementia
      3. Alzheimers dementia
         1. Alzheimers
         2. Alzheimer’s disease
         3. Alzheimers disease
         4. Alzheimer’s dementia
         5. Alzheimers dementia
      4. Vascular dementia
         1. Vascular dementia
         2. Binswanger’s disease
         3. Binswangers disease
         4. Binswanger disease
         5. Mixed Alzheimers-vascular dementia
         6. Mixed vascular-Alzheimers dementia
2. Conditions impeding assessment of treatment effect
   1. Stroke, unspecified
      1. Stroke NOS
      2. Acute Stroke NOS
      3. Cerebrovascular accident
      4. Acute cerebrovascular accident
      5. CVA
      6. Acute CVA
      7. Chronic CVA
      8. Prior CVA
      9. Previous CVA
      10. Old CVA
   2. Intracranial hemorrhage
      1. Epidural hemorrhage
      2. EDH
      3. Subdural hemorrhage
      4. SDH
      5. Subarachnoid hemorrhage
      6. SAH
      7. Intracerebral hemorrhage
      8. ICH
      9. Intraparenchymal hemorrhage
      10. IPH
      11. Intraventricular hemorrhage
      12. IVH
   3. Other symptomatic intracranial pathologies
      1. Anoxic brain injury
         1. Hypoxic-ischemic brain injury
         2. Brain anoxia
         3. Brain anoxic injury
      2. Brain tumor
         1. Glioma
         2. Astrocytoma
         3. CNS lymphoma
         4. Medulloblastoma
         5. Oligodendroglioma
         6. Brain met
         7. Brain metastasis
      3. Brain abscess and other intracranial infections
         1. Brain abscess
         2. Toxoplasma
         3. Toxoplasmosis
         4. Neurocysticercosis
         5. Encephalitis
         6. Meningoencephalitis
         7. Encephalomyelitis
         8. Progressive multifocal leukoencephalopathy
         9. PML
         10. CNS Lyme
         11. Neuroborreliosis
         12. Rabies
         13. Creutzfeldt-Jakob disease
         14. CJD
         15. Prion disease
      4. Multiple sclerosis
         1. Relapsing remitting multiple sclerosis
         2. RRMS
         3. Secondary progressive multiple sclerosis
         4. SPMS
      5. Contusion
         1. Brain contusion
         2. Cerebral contusion
         3. Hemorrhagic contusion
      6. Hydrocephalus
         1. Hydrocephalus
         2. Communicating hydrocephalus
         3. Obstructive hydrocephalus
   4. Other forms of dementia
      1. Pick’s disease
      2. Frontotemporal dementia
      3. FTD
      4. Normal pressure hydrocephalus
      5. NPH
      6. Dementia with Lewy bodies
      7. Lewy body disease
      8. Lewy body dementia
      9. LBD
      10. Corticobasal degeneration
      11. CBD
