# Supplementary material for: Assessment of the impact of EHR heterogeneity for clinical research through a case study of silent brain infarction
Source: BMC Med Inform Decis Mak. 2020 Mar 30;20:60. doi: 10.1186/s12911-020-1072-9 (PMC7106829; doi:10.1186/s12911-020-1072-9)
Supplement: Supplementary file 2 — Additional file 2: Supplemental Appendix 2. Annotation Guideline. [file 12911_2020_1072_MOESM2_ESM.docx]

**SUPPLEMENTAL** **APPENDIX 2. Annotation Guideline**

**Background** Silent brain infarction (SBI) is a brain lesion presumed to be due to vascular occlusion. Despite its high prevalence and serious consequences, little is known about the appropriate management of SBI since screening is not performed in routine care and would be a prohibitively expensive case-finding approach for a trial. In addition to SBI, white matter disease (WMD) or leukoaraiosis is another common finding in neuroimaging of elderly. Similar to SBI, WMD is usually detected incidentally on brain scans and is commonly believed to be a form of microvascular ischemic brain damage resulting from typical cardiovascular risk factors. Our goal is to systematically annotation findings of SBI and WMD in neuroimaging reports and neuroimages (Fig 1).

**Fig. 1** Example of neuroimaging report annotation (left) and neuroimages annotation (right) on SBI (yellow) and WMD (blue)

**Task 1: Neuroimaging Annotation Guidelines**

Revision 10.21.19

**1.1 Annotation Tool**

The annotation tool for this project is the Multi-document Annotation Environment (MAE), a Java-based natural language annotation software package. MAE is a non-web-based annotation tool. All annotation tasks will be defined in a document type definition (DTD) file. Due to its lightweight feature, the software can be easily shared and updated across multiple sites without configuration and testing.

Application: MAE_v0.9.6.jar

DTD version: sbi_07.dtd

Download: https://github.com/keighrim/mae-annotation

Annotators will be given access to a sample of clinical notes with .txt file format. After opening a .txt file in the MAE tool and allowing MAE to convert the file to an .xml format, annotators will be tasked with two primary goals. First, annotators will verify whether or not the patient meets the criteria for “SBI”. Second, annotators will identify and highlight keywords or phrases pertaining to the SBI and WMD (See 1.5 - 1.6)

**1. 2 Instruction**

1. Markup text for the same neuroimaging finding (i.e. SBI, WMD lesion) in **both** the body (Findings) and summary (Impression, Assessment) sections of the report. Highlight them **separately** (the annotations may not match up perfectly if the reporting of findings is inconsistent) (Fig 1).
2. If a field is left blank, that implies “not specified.”
3. “Negation” refers to the presence of a negative statement indicating the absence of a finding (i.e. a double negative).
   1. Example: “There is no evidence of acute infarction” would be annotated as SBI_found -> Acuity/Acute + Negation/Yes
4. If there is no mention of terms or synonyms suggesting infarction or white matter disease, do not annotate anything. Save the report as an XML to indicate that the report was reviewed.
5. Redacted, corrected, or “strike through” text in the neuroimaging reports should not be annotated.

**Fig 1.** Example of Annotating of SBI and WMD Findings in MAE


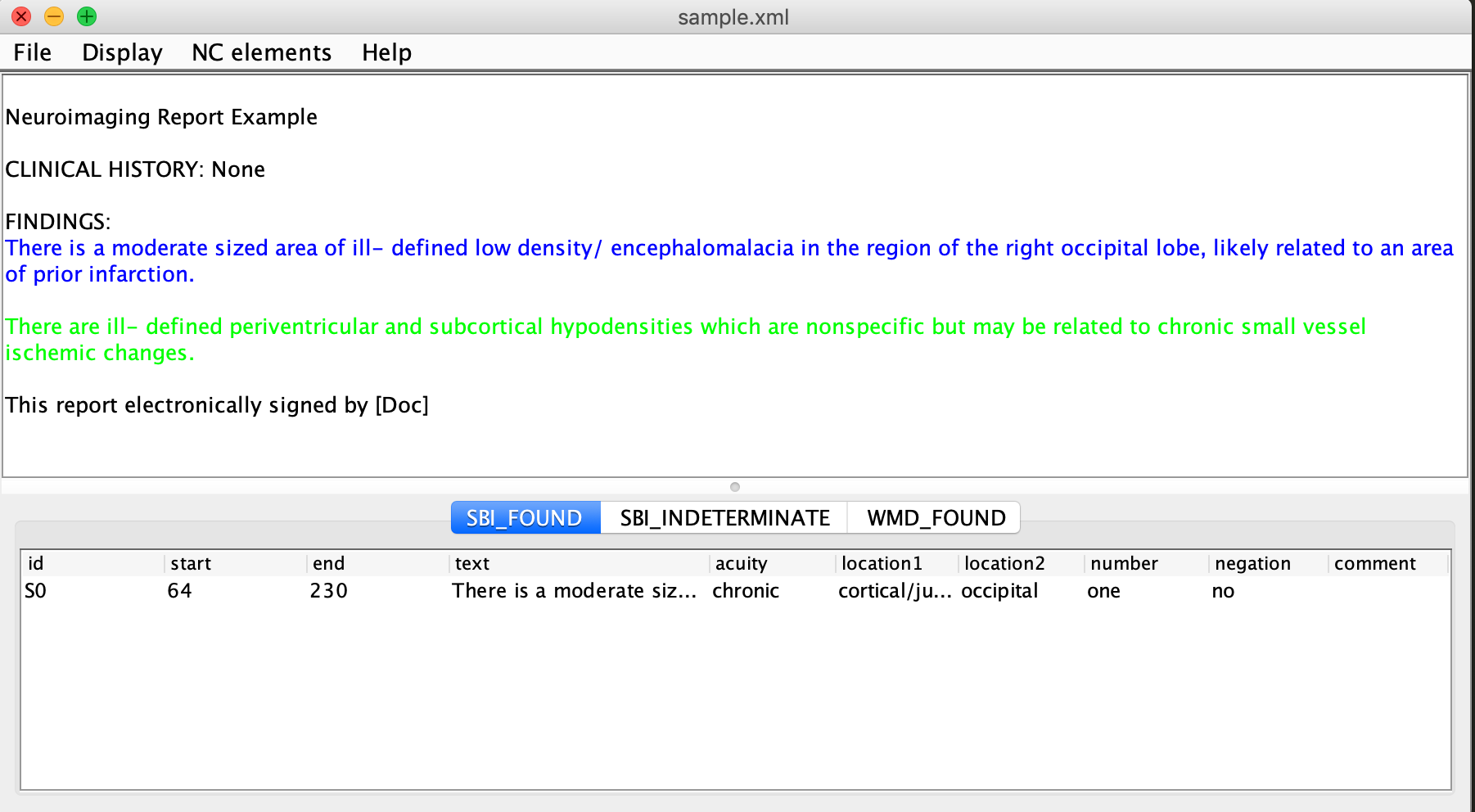


**1.3 Excluded neuroimaging study types**

1. MR angiogram of the head (without an accompanying MRI head)
2. CT sinus (low dose, insufficient resolution of the brain)
3. Cerebral angiogram (i.e. digital subtraction angiogram, conventional angiogram, catheter angiogram)

**1.4 Template hierarchy**

1. SBI_found
   1. Acuity
      1. acute/subacute
      2. chronic
      3. both
      4. not specified
   2. Location1
      1. lacunar/subcortical
      2. cortical/juxtacortical
      3. both
      4. not specified
   3. Location2
      1. frontal
      2. parietal
      3. temporal
      4. occipital
      5. insular
      6. basal ganglia
      7. caudate
      8. putamen
      9. globus pallidus
      10. thalamus
      11. brainstem
      12. cerebellum
      13. more than one
      14. not specified
   4. Number
      1. one
      2. two or more
      3. not specified
   5. Negation
      1. yes
      2. no
   6. Comment
2. SBI_indeterminate (use for indeterminate findings, such as “possible” SBI or WMD lesions)
   1. Type
      1. encephalomalacia
      2. perivascular space
      3. possible lacune (lacunar infarct)
      4. other
   2. Negation
      1. yes
      2. no
3. WMD_found
   1. Grade
      1. mild
      2. mild/moderate
      3. moderate
      4. moderate/severe
      5. severe
      6. no mention of quantification
   2. Negation
      1. yes
      2. no

**1.5 Language related to brain infarcts**

| **Category** | **Term** | **Suggested Category** | Example |
| --- | --- | --- | --- |
| Synonyms for infarct | Foci of restricted diffusion, restricted water diffusion, restricted diffusivity, diffusion restriction | Negation -> No  (i.e. infarct present) | “No focal masses, focal atrophy, or foci of restricted water diffusion.”  “No restricted diffusion.” |
|  | Lacune | Negation -> No |  |
| Certainty of infarct | Probable, likely | Negation -> No | T505: “which is likely secondary to a subacute infarct.” |
|  | Possible (or ambiguous language) | Annotate under SBI_indeterminate | “Prominent perivascular space or chronic lacunar infarct” |
|  | Encephalomalacia (not otherwise specified) | Annotate under SBI_indeterminate | T555: “Loss of gray-white matter differentiation and volume loss within the left middle frontal gyrus is consistent with an area of encephalomalacia.” (No subsequent mention of the nature of this lesion.) |
| Acuity of infarct | Acute | Acute |  |
|  | Acute/subacute | Acute | KP129: “Small foci of acute/subacute brain infarct cannot be excluded based on this examination.” |
|  | Subacute | Acute |  |
|  | Subacute/chronic | Acute |  |
|  | Chronic | Chronic |  |
|  | Age indeterminate | Not specified |  |
| Location of infarct | Lacune | Subcortical | T513: “There are old lacunes in the bilateral basal ganglia…” |
|  | Gyrus, cortex | Cortical | T581: “Small old infarcts are seen in the pre-central gyrus of the right frontal lobe, right parietal cortex and right occipital cortex.” |
| Number of infarcts | Several | 2 or more | T518: “Several foci in the cerebellum, right more than left also reflect old infarcts.” |

**1.6 Language related to white matter disease/leukoaraiosis**

| **Category** | **Term** | **{term}** | **Suggested Category** | Example |
| --- | --- | --- | --- | --- |
| Synonyms for white matter disease | Chronic {ischemic, small vessel disease} | Microangiopathy, small vessel disease, microvascular disease, small vessel ischemic disease, microvascular ischemic disease | Negation -> No  (i.e. WMD present) |  |
|  | Leukoaraiosis |  | Negation -> No |  |
|  | {} White matter change | Senescent, ischemic, age related, {blank} | Negation -> No | KP11: “A moderate degree of cerebral white matter changes is suspected.”  KP143: “Mild nonspecific ischemic and/or gliotic bihemispheric white matter changes, likely in part age related.” |
|  | Chronic ischemic and/or degenerative changes |  | Negation -> No |  |
|  | White matter {ischemic, small vessel} disease | Ischemic, microangiopathic, microvascular, small vessel | Negation -> No | KP128: “Inhomogeneity of white matter is seen due to white matter ischemic disease.” |
|  | {} White matter hypodensity or T2/FLAIR hyperintensity | Nonspecific, {blank, or no differential listed} | Negation -> No |  |
|  | White matter hypodensity or T2/FLAIR hyperintensity due to {} | {Long differential and no commitment to a “likely” diagnosis} | If microvascular or ischemic disease is mentioned -> Negation -> No  If microvascular or ischemic disease is not mentioned -> Do not annotate |  |
|  |  | {Short differential mentioning *chronic microvascular ischemic disease* and *migraines*} | Negation -> No |  |
| Quantification of white matter hyperintensities (MRI) or hypodensities (CT) | Few |  | Mild | T531: “There are a few scattered nonspecific foci of T2/FLAIR hyperintensity in the subcortical and periventricular white matter…” |
|  | Scattered |  | Mild | T505: “There are scattered foci and confluent areas of T2/FLAIR hyperintensity in the subcortical, deep and periventricular white matter as well as the central pons, a non-specific finding but likely reflecting the sequela of chronic microangiopathy.”  T571: “There are a few scattered foci of T2/FLAIR hyperintensity…”  T602: “There are mild scattered foci…” |
|  | Several |  | Moderate |  |
|  | Multiple |  | Moderate | T514: “There are multiple foci of T2/FLAIR hyperintensity…”  T581: “There are multiple scattered small foci and confluent areas of T2/FLAIR hyperintensity…” |
|  | Extensive |  | Severe | T613: “Extensive nonspecific bilateral T2/FLAIR hyperintensities…” |
|  | Diffuse |  | Severe | T597: “There are diffuse bilateral periventricular areas of T2/FLAIR hyperintensity…” |
|  | Mild-to-moderate, mild-moderate |  | Mild-moderate  (only use this category for this specific descriptor) |  |
|  | Moderate-to-severe, moderate-severe |  | Moderate-severe  (only use this category for this specific descriptor) |  |
| Topography/area of white matter hyperintensities (MRI) or hypodensities (CT) | Punctate |  | No influence on annotation | T528: “There are punctate and more confluent foci of T2/FLAIR hyperintensity in the periventricular and subcortical white matter.” |
|  | Focal, foci |  | No influence on annotation | T503: “There are additional foci of T2/FLAIR signal hyperintensity within the subcortical and periventricular white matter with additional involvement of the central pons, which are nonspecific although are presumably on the basis of chronic small vessel ischemic disease.” |
|  | Confluent |  | No influence on annotation | T521: “Focal and confluent subcortical and periventricular T2 and FLAIR hyperintensity is noted…”  T605: “Focal and confluent periventricular and subcortical white matter hypodensities are noted in the bilateral cerebral hemispheres, which likely represent mild changes of chronic small vessel ischemic disease.” |

**Task 2 - Neuroimaging Research Grade Interpretation Guidelines**

Revision 1.22.17

**2.1 Annotation Tool**

Excel Template

**2.2 Concept Definition**

- Study ID
- SBI
  - Present
    - - No
      - Yes
      - Indeterminate
  - If SBI indeterminate, most likely diagnosis?
    - - [Free text]
  - Acuity
    - - acute/subacute
      - chronic
      - both
  - Location: Cortical vs Subcortical (analogous to Location1 from Task 1)
    - - lacunar/subcortical
      - cortical/juxtacortical
      - both
  - Location: Region (analogous to Location2 from Task 1)
    - - frontal
      - parietal
      - temporal
      - occipital
      - insular
      - caudate
      - putamen
      - globus pallidus
      - thalamus
      - brainstem
      - cerebellum
      - more than one
      - other
  - Number
    - - one
      - two or more
- WMD
  - Present
    - - No
      - Yes
      - Indeterminate
  - If WMD indeterminate, most likely diagnosis?
    - - [Free text]
  - Grade (Manolio grading scale)
    - - 0
      - 1
      - 2
      - 3
      - 4
      - 5
      - 6
      - 7
      - 8
      - 9
      - 10
- Comment – space for notes about cases, including mention of non-SBI, non-WMD findings

**2.3 Consensus Imaging Definitions**

Study population: age > 50

**2.3.1.** **Silent brain infarcts**

CT definition (from the R01 grant): Discrete, focal hypodense lesion > 3 mm in size conforming to a vascular distribution in the white matter, gray matter, or both

MRI definition (from the R01 grant): Discrete, focal T2 hyperintense lesion > 3 mm in size conforming to a vascular distribution in the white matter, gray matter, or both with at least one of the following additional criteria: corresponding T1 hypointensity, DWI positivity (with or without ADC darkness), or cavitation with an irregular margin

|  |  | Acute/subacute, cortical | Acute/subacute, subcortical | Chronic, cortical | Chronic, subcortical |
| --- | --- | --- | --- | --- | --- |
| CT | Diameter |  |  |  | 3-15 mm |
|  | Density | Hypo | Hypo | Hypo | Hypo |
| MRI | Diameter |  |  |  | 3-15 mm |
|  | DWI | Hyperintense | Hyperintense | Isointense or Hypointense | Isointense or Hypointense |
|  | ADC | Hypointense (within 4-7 days) | Hypointense (within 4-7 days) | Hyperintense | Hyperintense |
|  | FLAIR | Hyperintense | Hyperintense | Hyperintense | Hyperintense |
|  | T2 | Hyperintense, no rim (homogeneous) | Hyperintense, no rim (homogeneous) | Hyperintense, ragged rim | Hyperintense, ragged rim |
|  | T1 | Hypointense | Hypointense | Hypointense | Hypointense |

**2.3.2.** **White matter disease/leukoaraiosis** (possible or probable vascular etiology)

CT definition (from the R01 grant): Confluent or poorly marginated hypodense lesions in the white matter.

MRI definition (from the R01 grant): Confluent or poorly marginated T2 hyperintensities involving the white matter that do not meet criteria for brain infarction.

|  |  | Chronic, subcortical |
| --- | --- | --- |
| CT | Diameter |  |
|  | Density | Hypo |
| MRI | Diameter |  |
|  | DWI | Isointense or Hypointense |
|  | ADC | Hyperintense |
|  | FLAIR | Hyperintense |
|  | T2 | Hyperintense |
|  | T1 | Hypointense |

**2.4 Examples of alternative imaging findings**

2.4.1. Perivascular spaces

|  |  | Chronic, subcortical |
| --- | --- | --- |
| CT | Diameter | ≤ 3 mm |
|  | Density | Hypo |
| MRI | Diameter | ≤ 3 mm |
|  | DWI | Hypointense |
|  | ADC | Hyperintense |
|  | FLAIR | Hypointense |
|  | T2 | Hyperintense, no rim |
|  | T1 | Hypointense |

2.4.2. Encephalomalacia (not due to ischemia, including trauma, post-surgical changes, degenerative brain atrophy, etc.)

|  |  | Chronic, cortical | Chronic, subcortical |
| --- | --- | --- | --- |
| CT | Diameter |  |  |
|  | Density | Hypo | Hypo |
| MRI | Diameter |  |  |
|  | DWI | Isointense or Hypointense | Hypointense |
|  | ADC | Hyperintense | Hyperintense |
|  | FLAIR | Hyperintense | Hypointense |
|  | T2 | Hyperintense, ragged rim | Hyperintense, no rim |
|  | T1 | Hypointense | Hypointense |

**References:**

1. Wardlaw JM, Smith EE, Biessels GJ, et al; STandards for ReportIng Vascular changes on nEuroimaging (STRIVE v1). Neuroimaging standards for research into small vessel disease and its contribution to ageing and neurodegeneration. Lancet Neurol. 2013;12:822–838.
2. Fanning JP, Wesley AJ, Wong AA, Fraser JF. Emerging spectra of silent brain infarction. Stroke. 2014;45:3461-3471.
3. Fu S, Leung LY, Wang Y, Raulli AO, Kallmes DF, Kinsman KA, Nelson KB, Clark MS, Luetmer PH, Kingsbury PR, Kent DM. Natural Language Processing for the Identification of Silent Brain Infarcts From Neuroimaging Reports. JMIR medical informatics. 2019;7(2):e12109.
