# Supplementary material for: Assessment of the impact of EHR heterogeneity for clinical research through a case study of silent brain infarction
Source: BMC Med Inform Decis Mak. 2020 Mar 30;20:60. doi: 10.1186/s12911-020-1072-9 (PMC7106829; doi:10.1186/s12911-020-1072-9)
Supplement: Supplementary file 3 — Additional file 3: Supplemental Appendix 3. Process Documentation TMC Manual Screening. [file 12911_2020_1072_MOESM3_ESM.docx]

**SUPPLEMENTAL APPENDIX 3. Process Documentation TMC Manual Screening**

Step 1: Obtain all Tufts scans with six CPT codes (CT: 70450, 70460 and 70470, MRI: 70551, 70552 and 70553) between 10/1/09-9/30/15 = 63,419 scans

Step 2: De-duplicate and perform initial SAS-based exclusion by ICD9 exclusionary codes (JN) = 12,092 remaining

Step 3: Screen by EMR electronically (eCW, Logician, Soarian) by problem lists for ICD9 codes and merge the results, and remove all patient-scan pairs that do not exist in triplicate (TW)

Step 4: Randomly select 1000 cases for manual screening (BK) = 1000 remaining

Step 5: Manual screening of Soarian discharge summaries by primary diagnosis, PMH, and hospital course (TW, SG, LYL ) = 907 remaining

Step 6: Extract neuroimaging reports (TW, SG) = 907 remaining

Step 7: Manual screen of the neuroimaging requisitions by LYL = 773 remaining

**Workflow description**

At TMC, the cohort was derived from individuals with neuroimaging scans between 2009 (the initial year neuroimaging reports entered the electronic medical record) and October 2015 (the initiation of ICD10 codes). The first neuroimaging scan chronologically entered into the electronic medical record was selected for each individual. An age restriction was applied excluding individuals 50 years of age or younger at the time of the first neuroimaging scan. ICD9 billing codes associated with the neuroimaging scan were collected. Individuals with neuroimaging scans associated with exclusionary ICD9 codes were removed from the cohort. Categories of exclusionary ICD9 codes are described in Figure 1 and are listed in full in the Supplemental Appendix Table 1. Subsequently, the list of exclusionary ICD9 codes were compared against ICD9 codes for problem lists or billed diagnoses for clinical encounters in three electronic medical records at TMC: General Electric Logician (outpatient general medicine), eClinicalWorks (outpatient specialties including neurology and neurosurgery), and Cerner Soarian (all inpatient encounters). Individuals with exclusionary codes in any of the three electronic medical records between the inception of the electronic medical record and 30 days following the neuroimaging scan date were removed. Integrity of this screening process was manually verified through full text review of the electronic medical records (10 individuals per step, 40 individuals total) by a study vascular neurologist (LYL) to determine if individuals were included or excluded appropriately at each step. Finally, the electronic medical records of the remaining individuals underwent two levels of manual screening by research assistants (TW, SG, LS) and a study vascular neurologist (LYL), respectively, using a list of free text exclusion criteria associated with the exclusionary ICD9 codes: the first involved reviewing the full text of the discharge summaries in Cerner Soarian, if present, associated with the encounter during which the neuroimaging scan was obtained, and the second involved reviewing the neuroimaging scan indication in the neuroimaging report. Of the remaining individuals, 500 were randomly selected, and full text of the neuroimaging reports were extracted manually. All neuroimaging reports were de-identified.
